# Pharmaceutical intervention for adverse events improves quality of life in patients with cancer undergoing outpatient chemotherapy

**DOI:** 10.1186/s40780-022-00239-w

**Published:** 2022-03-02

**Authors:** Hironori Fujii, Yukino Ueda, Chiemi Hirose, Koichi Ohata, Kumiko Sekiya, Mika Kitahora, Shiori Sadaka, Senri Yamamoto, Daichi Watanabe, Hiroko Kato-Hayashi, Hirotoshi Iihara, Ryo Kobayashi, Miho Kaburaki, Nobuhisa Matsuhashi, Takao Takahashi, Akitaka Makiyama, Kazuhiro Yoshida, Hideki Hayashi, Akio Suzuki

**Affiliations:** 1 Department of Pharmacy, Gifu University Hospital, Yanagido 1-1, Gifu, 501-1194 Japan; 2 Laboratory of Pharmacy Practice and Social Science, Gifu Pharmaceutical University, Gifu, Japan; 3 Laboratory of Community Healthcare Pharmacy, Gifu Pharmaceutical University, Gifu, Japan; 4 Department of Surgical Oncology, Gifu University Graduate School of Medicine, Gifu, Japan

**Keywords:** Quality of life, Pharmaceutical intervention, Adverse events related outpatient cancer chemotherapy, EuroQol 5 Dimension5 level (EQ-5D-5L), Retrospective observational study

## Abstract

**Background:**

The effect of pharmaceutical intervention to treat adverse events on quality of life (QOL) in outpatients receiving cancer chemotherapy is unclear. We investigated whether pharmaceutical intervention provided by pharmacists in collaboration with physicians improves QOL with outpatient cancer chemotherapy.

**Methods:**

We conducted a single-center retrospective descriptive study of pharmaceutical intervention for patients receiving outpatient cancer chemotherapy at Gifu University Hospital between September 2017 and July 2020. We assessed patient QOL using the Japanese version of the EuroQol 5 Dimension5 Level (EQ-5D-5L). Adverse events were graded using the Common Terminology Criteria for Adverse Events (CTCAE) version 4.0. We compared the EQ-5D-5L utility value and incidence of grade 2 or higher adverse events before and after pharmaceutical intervention.

**Results:**

Our analysis included 151 patients who underwent 210 chemotherapy cycles. Pharmaceutical intervention significantly improved patients’ EQ-5D-5L utility values from 0.8197 to 0.8603 (*P* < 0.01). EQ-5D-5L utility values were significantly improved after pharmaceutical intervention for nausea and vomiting (pre-intervention 0.8145, post-intervention 0.8603, *P* = 0.016), peripheral neuropathy (pre-intervention 0.7798, post-intervention 0.7988, *P* = 0.032) and pain (pre-intervention 0.7625, post-intervention 0.8197, *P* = 0.035). Although not statistically significant, the incidence of grade 2 or higher adverse events, including nausea and vomiting, dermopathy, pain, oral mucositis, diarrhea and dysgeusia, tended to be lower post-intervention than pre-intervention.

**Conclusions:**

Pharmaceutical intervention by pharmacists in collaboration with physicians may improve QOL in patients undergoing outpatient cancer chemotherapy.

## Background

Cancer is a leading cause of death, and cancer incidence is increasing globally, with a corresponding increase in the number of patients receiving cancer chemotherapy [1, 2]. Historically, anticancer drug therapy was performed only in hospital settings, but advances in the development of supportive therapy and modifications to the healthcare environment to reduce medical costs have facilitated broad-scale outpatient cancer chemotherapy [3, 4]. Although quality of life (QOL) is higher in patients receiving outpatient cancer chemotherapy than in those treated in hospital [5], the decreased access to medical management places these patients at risk of worse outcomes for several adverse events associated with anticancer drug therapy [6, 7].

We previously reported a significant correlation between adverse events, such as peripheral neuropathy, malaise and edema of the limbs, and decreased QOL, regardless of type of cancer or anticancer drug [8]. Similarly, Blanca Prieto-Callejero et al. reported that adverse events, such as nausea, dysgeusia, peripheral neuropathy and loss of appetite have a negative impact on QOL [9]. Iwamoto et al. reported severe mucositis/stomatitis affected health-related QOL in patients treated with cetuximab plus standard chemotherapy for advanced/metastatic colorectal cancer [10]. Moreover, Mol et al. found that patients with advanced-stage lung cancer who experienced strong negative feelings regarding the side effects of chemotherapy also showed decreased QOL, and recommended that physicians facilitate vigorous management of low-grade adverse events to improve QOL [11]. These reports highlight the necessity of reducing adverse events to enhance QOL. In turn, medical staff, including pharmacists, should therefore conduct monitoring and management for patients receiving outpatient-cancer chemotherapy. Pharmacists have the important role of selecting the appropriate drug therapy and contributing to adverse event reduction and supportive care by communicating with patients receiving cancer chemotherapy through patient interviews [12–14]. Pharmaceutical interventions have been shown to improve QOL in outpatients receiving cancer chemotherapy [15–17]. Although some studies have reported that patient education conducted by pharmacists had a positive impact, the effect of pharmaceutical intervention provided in collaboration with physicians to manage adverse events has not been evaluated.

Here, we investigated whether pharmaceutical intervention provided by pharmacists in collaboration with physicians improved QOL in outpatient cancer chemotherapy.

## Methods

### Study design

This retrospective observational study was conducted at Gifu University Hospital. Patients who underwent outpatient cancer chemotherapy between September 2017 and July 2020 were enrolled. We extracted utility values of QOL, adverse events, pharmaceutical intervention for adverse events, chemotherapy regimens, and other patient demographics from electronic medical records. Patients who underwent pharmaceutical intervention by pharmacists in collaboration with physicians for adverse events were included. Patients with missing or incomplete pre- or post-intervention QOL assessments and patients whose anticancer drugs were reduced by a physician were excluded.

### Assessment of QOL

We used EuroQol 5 Dimension 5 Level (EQ-5D-5L) to assess health-related QOL in patients undergoing outpatient cancer chemotherapy. The EQ-5D-5L questionnaire is a generic health status measure developed by the EuroQol group [18] to calculate quality-adjusted life years [19]. We used the Japanese version of the EQ-5D-5L, which was developed by Shiroiwa et al. in 2015 [20].

The questionnaire consists of five dimensions: mobility, self-care, usual activities, pain/discomfort, and anxiety/depression. Each dimension has 5 levels: level 1, no problem; level 2, slight problem; level 3, moderate problem; level 4, severe problem; level 5, unable or extreme [18]. A utility value ranging from 0 to 1 is calculated from the EQ-5D-5L, in which 0 indicates death and 1 indicates full health [21].

Patients who underwent outpatient cancer chemotherapy were asked to answer the EQ-5D-5L questions after each cycle. The Japanese version of the EQ-5D-5L questionnaire was used in face-to-face interviews to estimate the utility values of QOL and was routinely implemented by pharmacists during each patient visit. The utility values were recorded in the hospital’s electronic medical records system.

### Assessment of adverse events

In the outpatient chemotherapy clinic, physicians, pharmacists and nurses collaborated to assess adverse events, such as nausea and vomiting, diarrhea, oral mucositis, dysgeusia, peripheral neuropathy, pain, malaise, alopecia, and skin disorders. The severity of each adverse event was graded according to the Common Terminology Criteria for Adverse Events (CTCAE) version 5.0 [22]. All patients were provided with a daily checklist to confirm their side effects on their first visit to the outpatient chemotherapy clinic. Using the checklist, patients recorded the occurrence of daily adverse events after chemotherapy. From the returned checklists and the results of the interviews, pharmacists, in collaboration with physicians, recorded the severity of adverse events in the electronic medical records system. Physicians and pharmacists provided pharmaceutical intervention based on clinical practice guidelines to treat moderate or severe adverse events occurring during cancer treatment, and the effect of the intervention on the particular adverse event was assessed at the next consultation.

### Effect of pharmaceutical care on adverse events

We evaluated changes in QOL in patients who developed adverse events at two points, namely the onset of the adverse event (pre-intervention) and after pharmaceutical intervention for the adverse events (post-intervention). The timing of pre-intervention was the patient visit after the onset of each adverse event. The timing of post-intervention was the next patient visit after pharmaceutical intervention.

### Statistical analysis

We used IBM SPSS version 22 (IBM Japan Ltd., Tokyo, Japan) to analyze data. *P*-values < 0.05 were considered statistically significant. We summarized continuous variables using medians with 25th and 75th percentiles and categorical variables using frequencies and percentages.

The Wilcoxon signed-rank test was conducted to assess the effect of pharmaceutical interventions on QOL in patients experiencing adverse events. We compared the incidence of grade 2 or higher adverse events between pre- and post-intervention using McNemar’s test.

## Results

### Patients

**Fig. 1:**
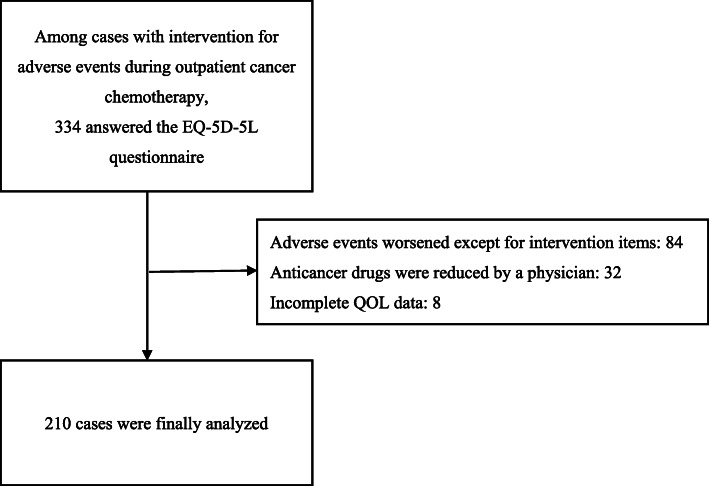
CONSORT diagram

Among patients who received pharmaceutical intervention for adverse events between September 2017 and July 2020, QOL was measured using EQ-5D-5L in 334 cases. Among these, we excluded 84 cases that were exacerbated by adverse events unrelated to the intended intervention, 32 cases whose anticancer drug doses were reduced by a physician, and 8 cases that did not have complete QOL data. We included 210 interventions (151 patients) in the analysis (Fig. 1). Patient characteristics are summarized in Table 1. The EQ-5D-5L questionnaire was obtained from 151 patients who received pharmaceutical intervention for adverse events. The most common type of cancer was breast cancer (27.8%), followed by colorectal (26.5%), pancreatic (13.2%), gastric (11.3%), head and neck (6.0%), and lung (6.0%). The most common type of regimen was oxaliplatin-based chemotherapy (21.2%), followed by paclitaxel/nanoparticle albumin-bound paclitaxel-based chemotherapy (17.9%), anthracyclines + cyclophosphamide (11.3%), gemcitabine + nanoparticle albumin-bound paclitaxel (7.9%), and irinotecan-based chemotherapy (7.3%).

**Table 1:** Patient demographics

Number of patients (male/female)	151	(63/88)
Age, median (min-max)	60	(28–82)
Number of chemotherapy courses	210	
Cancer		
Breast cancer	42	27.8%
Colorectal cancer	40	26.5%
Pancreatic cancer	20	13.2%
Gastric cancer	17	11.3%
Head and neck cancer	9	6.0%
Lung cancer	9	6.0%
Ovarian cancer/cervical cancer/uterine cancer	5	3.3%
Biliary tract cancer	4	2.6%
Angiosarcoma	1	0.7%
Esophageal cancer	1	0.7%
Malignant lymphoma	1	0.7%
Malignant pleural mesothelioma	1	0.7%
Peritoneal cancer	1	0.7%
Regimen		
L-OHP + fluoropyrimidines±Bmab/Pmab/Tmab	32	21.2%
PTX/Nab-PTX ± Tmab/ramucirumab/Bmab/Cmab	27	17.9%
Anthracyclines+cyclophosphamide	17	11.3%
GEM+Nab-PTX	12	7.9%
CPT-11 ± fluoropyrimidines±Bmab/aflibercept/ramucirumab/Pmab	11	7.3%
DOC/GEM/EPI/VNR/NabPTX+PER+Tmab	10	6.6%
FOLFIRINOX/FOLFOXIRI+Bmab	8	5.3%
CBDCA±PEM ± Bmab±Cmab	7	4.6%
GEM±S-1 ± CDDP	7	4.6%
Pembrolizumab/nivolumab/durvalmab/atezolizumab	6	4.0%
TAS102 + Bmab	4	2.6%
DOC/LipoDOX±Bmab	3	2.0%
Maintenance chemotherapy (Tmab/rituximab)	2	1.3%
S-1 + DOC	2	1.3%
Other (CMF/Eribulin/T-DM1)	3	2.0%

L-OHP, oxaliplatin; Bmab, bevacizumab; Pmab, panitumumab; Tmab, trastuzumab; PTX, paclitaxel; Nab-PTX, nanoparticle albumin-bound paclitaxel; Cmab, cetuximab; GEM, gemcitabine; CPT-11, irinotecan; DOC, docetaxel; EPI, epirubicin; VNR, vinorelbine; PER, pertuzumab; FOLFIRINOX/FOLFOXIRI, L-OHP + CPT-11 + 5-FU; CBDCA, carboplatin; PEM, pemetrexed; S-1, tegafur+gimeracil+oteracil; CDDP, cisplatin; LipoDOX, doxorubicin liposomal; TAS102, trifluridine; CMF, cyclophosphamide+methotrexate+ 5-FU; T-DM1, trastuzumab emtansine

### Pharmaceutical intervention for adverse events

The pharmaceutical interventions for adverse events are shown in Table 2. The adverse event with the most interventions was nausea and vomiting (33.1%; 50/210), followed by peripheral neuropathy (23.8%; 36/210), skin disorder (20.5%; 31/210), pain (19.2%; 29/210), and oral mucositis (16.6%; 25/210). The most common intervention for nausea and vomiting was oral administration of a D2 receptor blocker (48.0%), followed by olanzapine (18.0%), and aprepitant (12.0%). For peripheral neuropathy, the most common intervention was oral administration of duloxetine (47.2%) followed by neuropathic pain-alleviating agents (38.9%), and cryotherapy (11.1%). External application of steroid cream (38.7%) was the most common intervention for skin disorders, followed by heparinoids (25.8%), and oral administration of H1 receptor blockers (12.9%). For pain, the most common intervention was oral administration of non-steroidal anti-inflammatory drugs (44.8%), followed by opioids (37.9%), and acetaminophen (24.1%).

**Table 2:** Pharmaceutical intervention for adverse events

Adverse event	Intervention	Number	Rate
Nausea and vomiting (*n* = 50)	D2 blocker	24	48.0%
	Olanzapine	9	18.0%
	Aprepitant	6	12.0%
	5-HT3 receptor antagonist	4	8.0%
	Proton-pump inhibitor (PPI)	3	6.0%
	Mirtazapine	2	4.0%
	Others (Camostat/Butylscopolamine/Dexamethasone)	3	6.0%
Peripheral neuropathy (*n* = 36)	Duloxetine	17	47.2%
	Pregabalin/Mirogabalin Cryotherapy	14	38.9%
	Dose reduction of oxaliplatin (from 85 mg/m2 to 65 mg/m2)	4	11.1%
		1	2.8%
Skin disorder (*n* = 31)	Steroid cream	12	38.7%
	Heparinoids	8	25.8%
	H1 receptor blocker	4	12.9%
	Antimicrobial agent	3	9.7%
	Pemirolast	3	9.7%
	Others (Posterisan® forte/Urea cream/Crotamiton)	3	9.7%
Pain (*n* = 29)	Non-steroidal anti-inflammatory drugs (NSAIDs)	13	44.8%
	Opioids	11	37.9%
	Acetaminophen	7	24.1%
	*Shakuyakukanzoto*^*a*^	1	3.4%
Oral mucositis (*n* = 25)	Sodium azulenesulfonate preparation	15	60.0%
	Steroid	7	28.0%
	Sodium alginate	2	8.0%
	Zinc preparation	2	8.0%
Diarrhea (*n* = 15)	Probiotics	9	60.0%
	Loperamide	4	26.7%
	Hangeshashinto	3	20.0%
	Aluminium silicate	2	13.3%
	Others (Butylscopolamine/Alubumin tannate)	2	13.3%
Dysgeusia (*n* = 12)	Zinc preparation	12	100.0%
Malaise (*n* = 6)	Tapering of dexamethasone	5	83.3%
	*Hochuekkito*^*a*^	1	16.7%
Edema limbs (*n* = 4)	*Goreisan*^*a*^	2	50.0%
	Azosemide	1	25.0%
	Change from pregabalin to duloxetine	1	25.0%
Constipation (*n* = 2)	Probiotics	1	50.0%
	Sennoside	1	50.0%

^*a*^*Shakuyakukanzoto* and *Hochuekkito* are traditional herbal medicines commonly used in North-East Asian countries

### Relationship between pharmaceutical intervention and EQ-5D-5L utility values

The median EQ-5D-5L utility value and proportion of patients scoring at level 2 or higher in each dimension pre- and post-intervention are shown in Table 3. Overall, EQ-5D-5L utility values significantly improved (0.8197 pre to 0.8603 post; *P* < 0.01) after pharmaceutical intervention for adverse events. A significant change in the proportion of patients with level 2 or higher scores pre- and post-intervention was observed only in the ‘mobility’ dimension (37.1% pre to 30.5% post; *P* = 0.014).

**Table 3:** Comparison of EuroQol 5 Dimension-5 Level (EQ-5D-5 L) utility values between pre- and post-intervention

	Pre-intervention	Post-intervention	*P* value
Utility value (IQR)	0.8197 (0.7096–0.8978)	0.8603 (0.7543–0.9384)	< 0.01
Dimension	Proportion of level ≥ 2 (%)		
Mobility	37.1	30.5	0.014
Personal care	14.3	13.3	0.804
Usual activities	41.9	38.1	0.185
Pain/discomfort	65.2	58.6	0.071
Anxiety/depression	45.7	41.0	0.144

210 cases were analyzed. The Wilcoxon signed-rank test was used to compare the EQ-5D-5L utility value before and after pharmaceutical care. The McNemar test was used to compare percentage of level ≥ 2 in each of 5 dimensions between pre-intervention and post-intervention

### Pre- and post-intervention changes in EQ-5D-5L utility values and incidence of grade 2 or higher adverse events

**Fig. 2:**
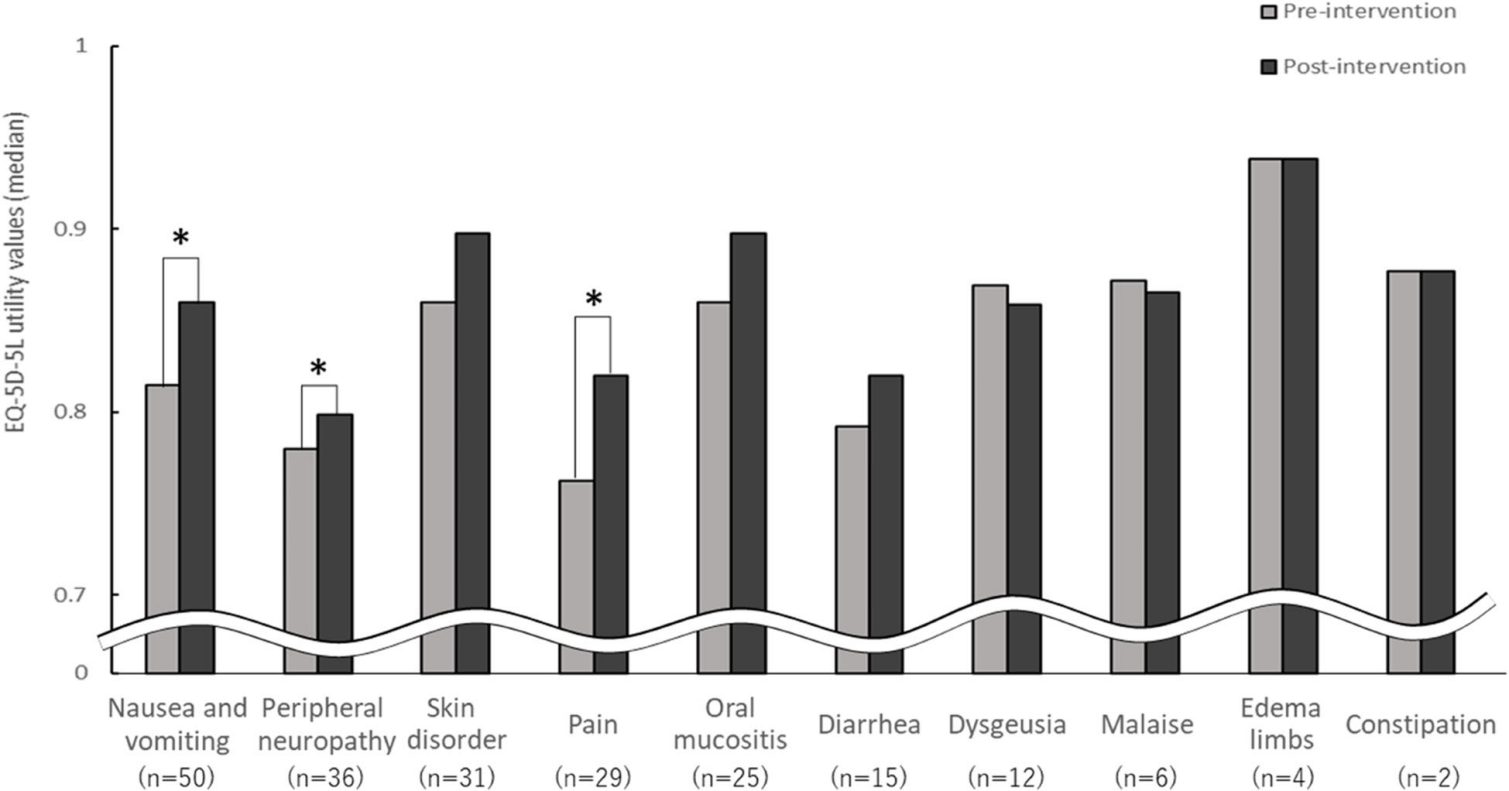
Comparison of median EQ-5D-5L utility values pre- and post-intervention for each adverse event. The Wilcoxon signed-rank test was used to assess differences (*P < 0.05). Pre-intervention, adverse event onset; post-intervention, after pharmaceutical intervention

Figure 2 shows the median EQ-5D-5L utility values pre- and post-intervention for each adverse event. EQ-5D-5L utility values significantly improved from pre- to post-intervention for nausea and vomiting (0.8145 pre to 0.8603 post; *P* = 0.016), peripheral neuropathy (0.7798 pre to 0.7988 post; *P* = 0.032), and pain (0.7625 pre to 0.8197 post; *P* = 0.035). Although not statistically significant, the median EQ-5D-5L utility values tended to increase for skin disorder (0.8603 pre to 0.8978 post; *P* = 0.057), oral mucositis (0.8603 pre to 0.8978 post; *P* = 0.121) and diarrhea (0.7923 pre to 0.8197 post; *P* = 0.328). Comparing the proportion of level 2 or higher scores in the 5 dimensions for each adverse event, there were no significant changes pre- and post-intervention (data not shown).

**Fig. 3:**
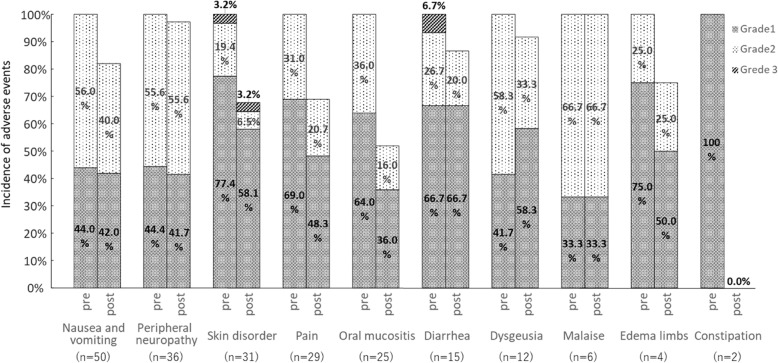
Comparison of adverse event incidence and grade pre- and post-intervention. McNemar’s test was used to compare the incidence of grade 2 or higher adverse events before and after pharmaceutical intervention

The incidence of adverse events is shown in Fig. 3. The incidence of grade 2 or higher nausea and vomiting (56.0% pre to 40.0% post; *P* = 0.18), skin disorder (22.6% pre to 9.7% post; *P* = 0.125), pain (31.0% pre to 20.7% post; *P* = 1.0), oral mucositis (36.0% pre to 16.0% post; *P* = 0.25), diarrhea (33.4% pre to 20.0% post; *P* = 1.0) and dysgeusia (58.3% pre to 33.3% post; *P* = 0.375) tended to be lower post-intervention, albeit that these differences were not significant.

## Discussion

In this study, we investigated the effects of pharmaceutical interventions for adverse events provided by pharmacists in collaboration with physicians on QOL in patients receiving outpatient cancer chemotherapy. Evaluation by EQ-5D-5L revealed that interventions were associated with a significant post-intervention improvement in patient QOL. In particular, interventions for nausea and vomiting, pain, and peripheral neuropathy were associated with significant improvements in QOL before and after the intervention. These results indicate that pharmaceutical intervention provided by pharmacists in collaboration with physicians for adverse events is useful for improving QOL in cancer chemotherapy.

Comparison of EQ-5D-5L utility values before and after pharmaceutical intervention for each of the 5 dimensions revealed that mobility was significantly improved post-intervention. The proportion of patients with a mobility level higher than 2 significantly decreased after pharmaceutical intervention. Similarly, a population-based cohort study of women diagnosed with breast cancer reported that patients with higher exercise metabolic equivalent scores had higher total QOL scores [23]. Eyl et al. also reported that QOL in long-term colorectal cancer survivors was higher in those who were more physically active than non-active survivors [24]. These findings indicate that improved mobility after the receipt of pharmaceutical intervention may contributed to enhancing QOL.

EQ-5D-5L utility values significantly increased after pharmaceutical intervention for nausea and vomiting. Hagiwara et al. showed that grade 1 nausea negatively impacted patient QOL [25]. Chemotherapy-induced nausea and vomiting are common side effects that can greatly impact patients receiving outpatient cancer-chemotherapy [26]. Additionally, the Clinical Practice Guidelines for Antiemesis from the Japan Society of Clinical Oncology highlight the importance of managing delayed nausea and vomiting during outpatient cancer-chemotherapy [27]. At Gifu University Hospital, pharmacists work in collaboration with physicians to select antiemetic agents in consideration of comorbidities and the appearance of nausea and vomiting after chemotherapy. Andrea et al. reported that antiemetic responses and patient satisfaction were significantly improved with the use of supportive medication prescribed according to evidence-based treatment guidelines and patient counseling for the management of treatment-associated adverse events [28]. Thus, individualized antiemetic treatment may contribute to improving patient QOL. Moreover, we found that EQ-5D-5L utility values were significantly improved when aprepitant was orally administered to treat nausea and vomiting (data not shown). Similarly, Thomas et al. reported that the addition of aprepitant to a standard regimen resulted in significantly less chemotherapy-induced nausea and vomiting and improved QOL [29].

Pharmaceutical interventions for pain also significantly improved EQ-5D-5L values. In particular, post-intervention EQ-5D-5L utility values significantly improved after opioids were added to the pain treatment regimen (data not shown). This finding is consistent with previous reports that QOL was improved after oral administration of opioids for severe pain in patients with cancer [30]. Clinical guidelines for cancer pain management suggest that opioids should be administered to cancer patients with moderate-to-severe pain [31–34]. The relationship between QOL and pain is inversely proportional in patients with breast cancer [35]. Therefore, the oral administration of opioids can relieve more severe pain and significantly improved patient QOL.

EQ-5D-5L utility values were also significantly improved after pharmaceutical intervention for peripheral neuropathy. Administration of pregabalin and duloxetine [36, 37] and the use of frozen gloves (cryotherapy) [38] are reported to improve QOL in patients with peripheral neuropathy. Although overall EQ-5D-5L utility values improved post-intervention, no significant differences were observed in the incidence of grade 2 or higher adverse events. According to the CTCAE, grade 2 peripheral neuropathy is a symptom that affects daily life, and the range of symptoms included in this classification is wide. In terms of the CTCAE assessment of peripheral neuropathy, pharmaceutical interventions were found to improve symptoms, which may have contributed to enhancing patient QOL. To assess the effect of pharmaceutical intervention for peripheral neuropathy, we considered that it would be necessary to use not only CTCAE but also NRS; however, we did not assess peripheral neuropathy using NRS or VAS in this study, and determining which scale is more appropriate for assessment of peripheral neuropathy is a question for future research.

In this study, we demonstrated that pharmaceutical intervention provided by pharmacists in collaboration with physicians contributed to QOL enhancement. Comprehensive pharmaceutical care increases patients’ chemotherapy-related knowledge, improves positive emotions, facilitates the management of chemotherapy-induced adverse events, and improves QOL [39]. An association between lower QOL and increased all-cause mortality has been reported [40]. Accordingly, to improve therapeutic effects, QOL should be maintained or enhanced in patients undergoing outpatient chemotherapy. The relationship between QOL enhancement and therapeutic effects warrants elucidation in future study.

Several limitations of this study should be acknowledged. First, because we were unable to compare our results with a control group that did not receive pharmaceutical intervention, it is unclear whether the improvement in QOL was due to pharmaceutical intervention or time. Second, the degree of cancer progression and the environment surrounding patients receiving outpatient-cancer chemotherapy can strongly impact QOL. We did not give sufficient consideration to patient condition, such as tumor metastasis, line of treatment, employment, and marital status. Third, it was not possible to clarify confounding factors such as differences among non-physician staff in charge (e.g., whether they were certified nurses or not). Furthermore, this study was a single-center study with a small sample size, which could result in exposure of the study population to a certain common bias. Further studies with a larger sample size are needed to verify our findings.

## Conclusion

This study suggests that the enhancement of supportive care for adverse events, such as nausea and vomiting, peripheral neuropathy, and pain, may improve the QOL of outpatients undergoing chemotherapy. Pharmaceutical intervention provided by pharmacists in collaboration with physicians is useful in maintaining and improving QOL and in the management of adverse events during cancer treatment.

## Data Availability

Not applicable.
